# Advances in RNA 3D Structure Modeling Using Experimental Data

**DOI:** 10.3389/fgene.2020.574485

**Published:** 2020-10-26

**Authors:** Bing Li, Yang Cao, Eric Westhof, Zhichao Miao

**Affiliations:** ^1^Center of Growth, Metabolism and Aging, Key Laboratory of Bio-Resource and Eco-Environment of Ministry of Education, College of Life Sciences, Sichuan University, Chengdu, China; ^2^Architecture et Réactivité de l’ARN, Institut de Biologie Moléculaire et Cellulaire du CNRS, Université de Strasbourg, Strasbourg, France; ^3^Translational Research Institute of Brain and Brain-Like Intelligence, Department of Anesthesiology, Shanghai Fourth People’s Hospital Affiliated to Tongji University School of Medicine, Shanghai, China; ^4^Newcastle Fibrosis Research Group, Institute of Cellular Medicine, Faculty of Medical Sciences, Newcastle University, Newcastle upon Tyne, United Kingdom; ^5^European Molecular Biology Laboratory, European Bioinformatics Institute (EMBL-EBI), Cambridge, United Kingdom

**Keywords:** RNA structure, chemical probing, 3D shape, structure prediction, RNA-puzzles

## Abstract

RNA is a unique bio-macromolecule that can both record genetic information and perform biological functions in a variety of molecular processes, including transcription, splicing, translation, and even regulating protein function. RNAs adopt specific three-dimensional conformations to enable their functions. Experimental determination of high-resolution RNA structures using x-ray crystallography is both laborious and demands expertise, thus, hindering our comprehension of RNA structural biology. The computational modeling of RNA structure was a milestone in the birth of bioinformatics. Although computational modeling has been greatly improved over the last decade showing many successful cases, the accuracy of such computational modeling is not only length-dependent but also varies according to the complexity of the structure. To increase credibility, various experimental data were integrated into computational modeling. In this review, we summarize the experiments that can be integrated into RNA structure modeling as well as the computational methods based on these experimental data. We also demonstrate how computational modeling can help the experimental determination of RNA structure. We highlight the recent advances in computational modeling which can offer reliable structure models using high-throughput experimental data.

## The Problem of RNA 3D Structure Determination and Its History

Ribonucleic acids or RNAs play significant roles in a great variety of biological processes throughout the central dogma (Sharp, 2009; Cech and Steitz, 2014; Strobel et al., 2016), ranging from transcription regulation (Long et al., 2017), to RNA splicing (Buratti and Baralle, 2004; Luco and Misteli, 2011), and protein synthesis (Valencia-Sanchez et al., 2006). A recent study revealed the fact that RNA may act as a riboregulator of autophagy through the regulation of protein polymerization (Horos et al., 2019), which indicates the function of the proteins are not only regulated by transcription and translation but also by interaction with the RNA structure. The functional diversity of RNA arises from its ability to form specific 3D structures that can function in response to cellular signals (Al-Hashimi and Walter, 2008; Mustoe et al., 2014, 2018). An example in disease mechanism from recent research (Cammas and Millevoi, 2017) shows RNA G-quadruplexes, folded into a four-stranded conformation, revealing new mechanisms in disease. According to these examples, determining and modeling the RNA structure can substantially contribute to our understanding of biological processes, disease mechanisms, and RNA therapies.

As early as 1969, the first manually predicted tertiary structure of tRNA (Levitt, 1969) was regarded as a milestone in the emergence of bioinformatics. That structure was based on available tRNA sequences and some scattered experimental data [like the cross-link between positions 8 and 13 (Yaniv et al., 1969)]. In 1989, the model of the core of group I intron (Michel and Westhof, 1990) was based on extensive sequence comparisons, clustering secondary structures into distinct classes, and the published experimental data based on mutagenesis. Since then, the RNA structure modeling approaches, including secondary and tertiary structure modeling, have been in intense development. A variety of useful programs were produced in this period [for recent reviews see (Miao and Westhof, 2017; Ponce-Salvatierra et al., 2019)]. With the development of whole-genome sequencing techniques and the availability of various metagenome sequences, hundreds of RNA families have been discovered (Weinberg et al., 2007, 2009, 2010) which greatly expanded our knowledge of the RNA sequence space. Almost all of these RNA sequence families have been described in the database of Rfam (Kalvari et al., 2018). However, up to January 2020, only 99 RNA families in the Rfam database have experimentally determined structures available in the Protein Data Bank (PDB) (Berman et al., 2000) or the Nucleic Acids Database (NDB) (Berman et al., 1992; Coimbatore Narayanan et al., 2014).

The experimental methods of RNA structure determination can be classified as biophysical and biochemical methods. Biophysical experiments such as x-ray crystallography (Suddala and Zhang, 2019), small-angle scattering (SAS) (Hura et al., 2009), and cryogenic electron microscopy (cryo-EM) (Zhang et al., 2019) are uncovering the structural basis of RNA functions at nanometer- or angstrom-level resolutions. Alternatively, biochemical approaches [e.g., chemical probing (Peattie and Herr, 1981)], have been used systematically to validate RNA structures. The recent coupling of RNA structure probing with high-throughput sequencing (Strobel et al., 2018) has changed the estimation from the electrophoresis on denaturing gels (Lucks et al., 2011) to the omics techniques, allowing higher for throughput in RNA structure characterization. Instead of the atomistic models, biochemical approaches promote the experimental flexibility and throughput by sacrificing the resolution. They determine RNA structure using the computational approaches based on the restraints obtained from experiments. Structure dynamics, folding, and *in vivo* structure determination have become a new field known as conformational ensembles, or structure ensembles. The ensembles are the set of all dynamic structure conformations, capturing the structure motions in a large range of energy landscapes and timescales. Conformational ensembles are critical in understanding the cellular function of RNAs (Ganser et al., 2019) and computational methods with experimental data have been developed (Salmon et al., 2014). And these methods have been discussed in previous reviews (Salmon et al., 2014; Ganser et al., 2019).

Together with the advances in experiments, computational modeling or prediction methods of RNA secondary and tertiary structure (Magnus et al., 2014; Ponce-Salvatierra et al., 2019) are being developed and improved to help and complement experimental efforts. Similar to the efforts in protein structure prediction, RNA 3D structure modeling has used approaches including homology modeling (Flores and Altman, 2010; Rother et al., 2011b), fragment assembly (Das and Baker, 2007; Bida and Maher, 2012; Popenda et al., 2012; Zhao et al., 2012; Xu et al., 2019), and *de novo* prediction (Sharma et al., 2008; Jonikas et al., 2009; Krokhotin et al., 2015; Boniecki et al., 2016). The direct coupling analysis approach (De Leonardis et al., 2015; Weinreb et al., 2016; Wang et al., 2017), which is based on the alignments of metagenome sequence information, also shows its ability in RNA structure prediction. Since 2011, RNA-Puzzles (Cruz et al., 2012; Miao et al., 2015, 2017, 2020), which is a community effort for evaluating these RNA 3D structure prediction methods, has reported three rounds of predictions. It revealed existing bottlenecks in RNA structure modeling, including the prediction of *non*-Watson–Crick interactions, atomic clashes in the models, and the challenges in ligand binding predictions.

The recent improvements about static structure determination, achieved by coupling high-throughput experimental data with computational modeling (Cheng et al., 2015, 2017; Kappel et al., 2019), have demonstrated great potential in RNA 3D structure characterization. In this review, we provide a concise overview of the existing experimental data in RNA structure determination and the structure modeling approaches. In particular, we highlight the recent achievements in experimental data-driven RNA structure modeling, which may revolutionize RNA structural biology in a high-throughput way in the near future.

## RNA Structure Characterization Experiments

**FIGURE 1:**
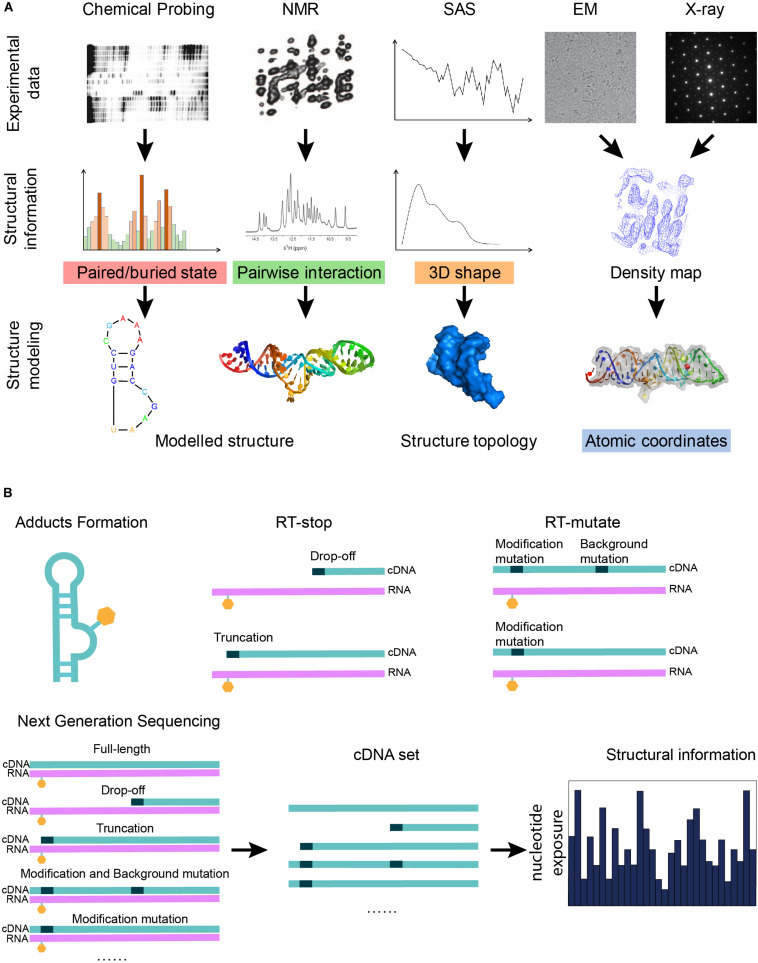
Graphical illustration of the experimental data that can be used in RNA structure modeling. **(A)** A graphical summary shows how structural information derived from biophysical and biochemical experiments can be used for structure modeling. Different experiments indicate different types of structural information: the three-dimensional shape of a molecule can be given by x-ray crystallography, cryo-EM, or SAS; pairwise interactions, including base-pair interactions and atomic contacts, are indicated from NMR or mutate-and-map; and features of a single nucleotide are inferred from chemical probing. **(B)** A scheme shows that the next-generation sequencing technique can be applied to the cDNA sets generated from the biochemical probing experiments in order to increase the throughput of the experiments. Chemical probing reagents modify the exposed nucleotides and result in adducts (orange hexagons). Adducts interrupt the reverse transcription known as RT-stop, while RT-mutate means that reverse transcription introduces a mismatched nucleotide (black dots) at the position of the adduct under special conditions. The sequencing results of the cDNA sets from RT-stop/RT-mutate can be transformed back to structural restraints.

Both biophysical and biochemical approaches (Figure 1A) have been conceived to determine RNA structures, dynamics, and interactions with other biomolecules. Biophysical experiments are normally used to generate 3D structural information in the form of shapes to describe the molecular structure, ranging from the angstrom-level methods of x-ray crystallography (Shi, 2014) and cryo-EM (Zhang et al., 2019) to the nanometer-level methods like SAS (Hura et al., 2009) and AFM (Pallesen et al., 2009). Biochemical approaches probe the RNA structures by generating the local structural features or restraints (Kubota et al., 2015) in computational modeling from early on (Westhof et al., 1989). Normally, biophysical experiments determine the structure of one RNA each time at the angstrom-level resolution, while biochemical can probe RNA structures in a high-throughput way by sacrificing the resolution. The well-established biophysical and biochemical approaches for RNA are introduced in the section below.

### Biophysics Approaches to Characterize RNA Structures

#### X-Ray Crystallography and Cryogenic Electron Microscopy (Cryo-EM)

X-ray crystallography and cryogenic electron microscopy (cryo-EM) approaches characterize RNA structures by 3D maps/density maps, which describe the shape of the molecule. In x-ray crystallography, crystalized samples are irradiated by x-ray from different angles to generate a group of diffraction data, which are interpreted into electron density maps after solving the phase problem by an inverse Fourier transform (Cate and Doudna, 2000). The phase problem in crystallography is normally solved by molecular replacement (Evans and McCoy, 2008), isomorphous replacement, anomalous dispersion (McCoy and Read, 2010), or their combination. The quality of a density map depends on the resolution of the structure as well as the thermodynamic mobility of the molecule. It is known that structural regions of high-temperature factors (B factors) may not have a clear electron density to infer the atomic coordinates (Magnus et al., 2020). Thus, computational modeling may optimize the crystal structures using structure knowledge learned from already solved structures (Terayama et al., 2018). Along with the recent advances in detector technology and software algorithms (Doerr, 2017), cryo-EM can be applied on purified macromolecule samples cooled to cryogenic temperatures and embedded in an environment of vitreous water, without the step of crystallization (Liao et al., 2013; Murata and Wolf, 2018; Vinayagam et al., 2018). Each image from cryo-EM shows a view of the macromolecule from a certain angle, while a massive number of particle images are used to reduce the noise and restructure the 3D shape of the molecule at an atomic resolution (Merk et al., 2016). In spite of the expensive equipment and the challenge in working with liquid samples, cryo-EM is now capable of solving a wide range of RNA structures with atomistic models. The determination of SAM-IV riboswitch demonstrates the capability of cryo-EM in solving RNA structures smaller than 40 kDa (Zhang et al., 2019), which is a breakthrough in solving small RNA molecules. Recent advances suggest that the combination of cryo-EM and x-ray crystallography can both solve the phase problem and determine the structure at high resolution (Wang and Wang, 2017). For both x-ray crystallography and cryo-EM, computational models are used to position the atoms into the experimentally obtained electron density.

#### Small-Angle Scattering (SAS) and Atomic Force Microscopy (AFM)

Small-angle scattering (SAS) and atomic force microscopy (AFM) are low-resolution techniques. SAS, consisting of the small-angle scattering of x-rays (SAXS) and neutrons (SANS), is capable of delivering structural information in the resolution range between 1 and 25 nm. Such information is similar to low-resolution cryo-EM data. SAS samples are dissolved in solution and are exposed to a beam of x-rays or neutrons. Subsequently, scattering data is collected from the detector. SAXS and SANS differ in their scattering particles: SAXS shows the electron density map while SANS shows the distribution of the nuclei of atoms (Byron and Gilbert, 2000; Koch et al., 2003; Svergun and Koch, 2003), both of which are used to determine the size and shape of particles (Schnablegger and Singh, 2011). Considering the resolution of SAS, it is only possible to capture the global shape of a macromolecule rather than structural details (Skou et al., 2014; Plumridge et al., 2018). In AFM experiments, samples are immobilized on a solid base and raster-scanned by an ultrasharp tip. The tip reacts with the sample in tapping or contact mode, causing the movement of the Piezo element. The movement-resulted laser reflection change can be detected by a photodetector. And the detected signal is then transformed into surface information (Sahin et al., 2007). The 3D surface images produced by AFM can reach a nanometer scale resolution (Shahin and Barrera, 2008), while computation is required to process these images to construct 3D structure models. Possible ways to improve AFM accuracy are better sample fixation and using a sharper tip (Schön, 2018). Successful examples of AFM determined RNA structures have been reported (Husale et al., 2009; Pallesen et al., 2009; Gilmore et al., 2014, 2017), while more details have been reviewed by Schön (2018).

#### Nuclear Magnetic Resonance (NMR) Spectroscopy

Nuclear magnetic resonance (NMR) spectroscopy, which covers ∼36% of the pure RNA structure solved in PDB, obtains RNA structural information from the chemical shift of the resonance frequencies of the nuclear spins in the sample. NMR is based on the physical observation that nuclei in a strong constant magnetic field, when perturbed by a weak oscillating magnetic field, produce an electromagnetic signal with a frequency characteristic of the magnetic field at the nucleus. Two-dimensional NMR methods (Chaloner, 1990) are used to detect couplings or connectivities between nuclei that are close to each other in space. Some local structural information can be inferred from the chemical shift spectra. Furthermore, long-range structural information can be probed by residual dipolar couplings caused by the presence of an aligning medium that interferes with the isotropic tumbling of a molecule (Marion, 2013). However, it is necessary to use structural assumptions and computational models to supplement local information when solving large structures. One significant advantage of NMR in solving RNA molecules is its ability in exploring non-canonical geometries, such as non-Watson–Crick base pairs (Hermann and Westhof, 1999), coaxial stacking (Lescoute, 2006), stem-loops, and pseudoknots (Westhof and Jaeger, 1992) which are key in RNA structure modeling. In addition, NMR may probe the ligand, such as proteins or drugs, binding to RNA by seeing which resonances are shifted upon the binding of the ligand (Wemmer, 1996). With the development of NMR technology, solid-state NMR and solution NMR become important tools to solve RNA structure. Solution-state NMR is reported to characterize RNA dynamics with atomic models, but the sample is limited in size (Bothe et al., 2011). Later, solid-state NMR has also been reported to be used in RNA structure determination in high resolution together with matched experimental methods. Moreover, there is no limitation in size of RNA and crystallization is not necessary (Marchanka et al., 2015). Some successful examples have been reported in both solution NMR (Davis et al., 2004; Latham et al., 2005; Keane et al., 2015; Orlovsky et al., 2020) and solid-state NMR (Marchanka et al., 2015, 2018; Huang et al., 2017; Yang and Wang, 2018).

#### Fluorescence Resonance Energy Transfer (FRET)

Fluorescence resonance energy transfer (FRET) is used when neither crystal nor solution samples are available. In FRET experiments, the fluorescent donor and the acceptor are attached to the samples, while the evaluated energy transmission by intermolecular dipole–dipole coupling stimulates fluorescent signals. The intensity is related to the distance between the donor and the acceptor (Tuschl et al., 1994; Stephenson et al., 2016). So, FRET is capable of determining long-range contact ranging from 10 to 80 Å (Millar, 1996; Sekar and Periasamy, 2003). Compared to FRET, single-molecule FRET (smFRET) can obtain detailed information of individual molecules, which can be used to understand the folding dynamics and conformation changes (Karunatilaka and Rueda, 2009; Holmstrom and Nesbitt, 2016; Stephenson et al., 2016; Manz et al., 2017). Recently, smFRET was used to probe DNA hairpin changes under different pressures and temperatures, which indicates that we may understand structure changes in different surroundings with smFRET (Sung and Nesbitt, 2020). A brief comparison among the biophysics methods is shown in Table 1.

**TABLE 1:** A comparison of biophysics approaches.

Biophysics methods	Advantages	Limitations
X-ray crystallography	Atomic resolution structure (Shi, 2014)	Difficulty in crystal preparation;
		Phasing problem
Cryo-EM	Near atomic resolution structure (Murata and Wolf, 2018);	Expensive equipment;
	Capable of solving macromolecule structures;	Difficulty in dealing with liquid sample
	Shows a cluster of conformations	
SAS	Easy to perform experiment;	Nanometer-level resolution (Svergun and Koch, 2003;
	Capable of dealing with large molecules;	Rambo and Tainer, 2013);
	Time efficient	Sample aggregation
AFM	Easy to perform experiment;	Nanometer- to molecular resolution result (Schön, 2018)
	Surface information	
NMR	Sample in solution condition;	Sample aggregation;
	Possible to probe the structural dynamics or invisible states;	Limited size of the structure
	Atomistic structure models (Bothe et al., 2011)	
FRET	Easy to perform experiment;	Can get limited structure information;
	Angstrom-level resolution (Sekar and Periasamy, 2003)	Limited to small RNA structures;
		The signal-to-noise ratio

### Biochemical Approaches to Probe RNA Structures

Unlike biophysical approaches, biochemical approaches establish biochemical probing as a quantitative measurement of the RNA conformation, in particular, the base pair interactions, the base exposure, and the structural flexibility. Both secondary structure information and tertiary contact information can be obtained through biochemical approaches. Such information can be transformed into structural restraints to direct structure modeling. Experiments related to chemical probing are listed below:

#### Chemical Probing for Secondary Structure

##### Chemical probing

Chemical probing, which dates back to the 1980s (Peattie and Herr, 1981), detects structural information by introducing chemical modifications and changes to RNA using chemicals (Figure 1B) (Weeks, 2010; Kubota et al., 2015), whose reactivity depends on local RNA structure. These chemical reagents include dimethyl sulfate (DMS) (Tijerina et al., 2007; Homan et al., 2014a), silyl derivative *N*,*N*-(dimethylamino) dimethyl chlorosilane (DMAS-Cl) (Mortimer et al., 2009), carbodiimides (CMCT), kethoxal (Ehresmann et al., 1987), and glyoxal and its derivatives (Mitchell et al., 2018). Different reagents can react with specific sites or structures to form covalent adducts (Busan et al., 2019) at the modification sites, while unpaired nucleotides are more exposed and more inclined to be modified.

After the reaction between the probing reagents and the structured RNA, two methods can detect the modification of the RNA. For the first method, RNA is labeled before the modification can be further treated after the modification to form a strand scission, which can be directly detected by electrophoresis (Ehresmann et al., 1987). In another way, the RNA is reverse transcribed using a reverse transcriptase into a DNA copy. Reverse transcription with stop (RT-stop) or mutation (RT-mutate) is a common method to detect modifications. RT-stop (Figure 1B) (Brunel and Romby, 2000; Merino et al., 2005) is based on the reverse transcription interruption by the adducts, while RT-mutate (Figure 1B) is based on the fact that reverse transcription introduces a mismatched nucleotide at the position of the adduct under special conditions (Siegfried et al., 2014). RT-stop results in a pool of DNA truncations of different lengths, whose frequencies reflect the RNA structure profile and can be assayed on a gel, while RT-mutate results in a pool of cDNAs of different sequences which need to be profiled by high-throughput sequencing (HTS). Besides local structure blocking, nucleotide positions can also be protected by a binding protein (Smola et al., 2015a).

Several other recently proposed chemical probing techniques are introduced below. (1) Using bifunctional reagents to probe long-range contacts (Weeks, 2010); (2) Using mutational profiling (MaP) to reveal the dynamic states and reactive sites of an RNA (Homan et al., 2014a; Krokhotin et al., 2017); (3) Using 2′-Hydroxyl molecular interference (HMX) to identify tertiary interactions in the highly packed regions (Homan et al., 2014b); and (4) Using hydroxyl radical probing (HRP) to study RNA folding and direct structure refinement (Ding et al., 2012; Costa and Monachello, 2014). Simultaneously, computational methods are being developed to cooperate with the experimental developments, e.g., discrete molecular dynamics simulations (DMD) were used to produce 3D models using HMX data (Homan et al., 2014b).

##### In-line probing and enzymatic probing

In-line probing and enzymatic probing have similar principles to chemical probing but differ in their reactions. Enzymatic probing (Wan et al., 2013) is based on the fact that RNase enzymes, which are local structure-specific enzymes, cut different regions of an RNA molecule. Thus, the RNA structure can be probed using the enzyme cut truncations. In-line probing utilizes the feature that the 2′-hydroxyl reacts with the backbone phosphate group in some conditions (the 2′-hydroxyl and the phosphate group form an angle of 180 degrees, i.e., a ‘line’ structure required) to break the backbone. As flexible RNA nucleotides are more inclined to fulfill such a condition, in-line probing tests the local structure flexibility (Regulski and Breaker, 2008).

#### Chemical Probing for Secondary and Tertiary Information

Many chemical probing experiments are used to measure secondary structure information, while some reagents and methods may infer tertiary structure information. Experiments using bifunctional reagents (Weeks, 2010) and M2-seq (Cheng et al., 2017) were reported to reveal long-range contacts (Weeks, 2010). Moreover, HMX (Homan et al., 2014b), MOHCA (Das et al., 2008), RING-MaP (Krokhotin et al., 2017) experiments, and comparing SHAPE results achieved by different reagents (Steen et al., 2012) were also used to measure proximal tertiary interactions. Those examples show great potential in probing tertiary structure.

A widely used and further explored chemical probing method, selective hydroxyl acylation analyzed by primer extension (SHAPE) (Wilkinson et al., 2006; Spitale et al., 2013; Siegfried et al., 2014; McGinnis et al., 2015; Smola et al., 2015b; Lee et al., 2017; Mustoe et al., 2018; Smola and Weeks, 2018; Busan et al., 2019), is based on the acrylate reaction between electrophilic reagents and active RNA 2’-hydroxyl groups which always appear in the single-strand region (Wilkinson et al., 2006). It reports on RNA structure at a single nucleotide resolution, thus is used to generate highly accurate secondary structure models (Deigan et al., 2009). It can be combined with high-throughput sequencing by barcode based multiplexing (Lucks et al., 2011) and can use a combination of chemical probing reagents and experimental data (Kladwang et al., 2011b). SHAPE has been used to analyze large RNA structures, including the SARS-CoV-2 genome (Manfredonia et al., 2020) and the HIV-1 genome (Watts et al., 2009).

Recent advances in chemical probing lie in the enlarged throughput (Kwok et al., 2015) (probing many RNAs simultaneously) and the *in vivo* probing of RNA molecules in their cellular environment (Kubota et al., 2015; Nguyen et al., 2016). As for modification detection methods, some studies show that combining RT-mutate and RT-stop can mitigate bias and have a better insight into the structure since they can provide complementary information (Novoa et al., 2017; Sexton et al., 2017). A more recent study of mutational profiling (MaP) shows the advantages of RT-mutate in several aspects: RT-mutate is simpler and faster (Busan et al., 2019), while it works on long and complex RNAs (Siegfried et al., 2014; Smola et al., 2015b; Busan and Weeks, 2018). Additionally, RT-mutate provides relative adduct frequencies inferred from read-depth, which measures the reactivities of different segments in the same experiment (Mustoe et al., 2018).

Progress has also been made in regents exploration and the assessments of the SHAPE method (Lee et al., 2017; Busan et al., 2019). Five regents 1M7 (1-methyl-7-nitroisatoic anhydride), 1M6 (1-methyl-6-nitroisatoic anhydride), NMIA (*N*-methylisatoic anhydride), NAI (2-methylnicotinic acid imidazolide), and 5NIA (5-nitroisatoic anhydride) have been assessed and recommended to use in experiments of different conditions [94]. Moreover, to explore the correlation between RNA structure and its SHAPE result, a computational model named 3D structure-SHAPE relationship (3DSSR) was developed to generate the SHAPE profile for a given RNA structure. This model can also indicate the inconsistency between the structure model and the SHAPE data and thus exclude the unreasonable structure models [152].

#### Chemical Probing Coupled With Mutagenesis

Chemical probing (or also known as chemical mapping) and related methods generally probe the features, e.g., the pair/unpair state, of a single nucleotide. To understand the base-pair connection between nucleotides, chemical mapping is coupled with mutagenesis. If a base-pair forming nucleotide is mutated, its partner may become unpaired, and thus tends to become more exposed and more detectable by chemical mapping (Kladwang and Das, 2010; Kladwang et al., 2011a; Cordero and Das, 2015). Early examples have been shown from the group I intron (Duncan and Weeks, 2008) and tetrahymena ribozyme (Pyle et al., 1992) studies. Of course, some mutations may lead to significant perturbations to the structure, even the unfolding of an entire helix. In general, the method of coupling chemical mapping and mutagenesis, known as mutate-and-map or *M*^2^, achieves a highly accurate detection of the canonical base pairs (Kladwang et al., 2011a; Cheng et al., 2017), with ∼2% error rate in the helix region (Kladwang et al., 2011a).

**FIGURE 2:**
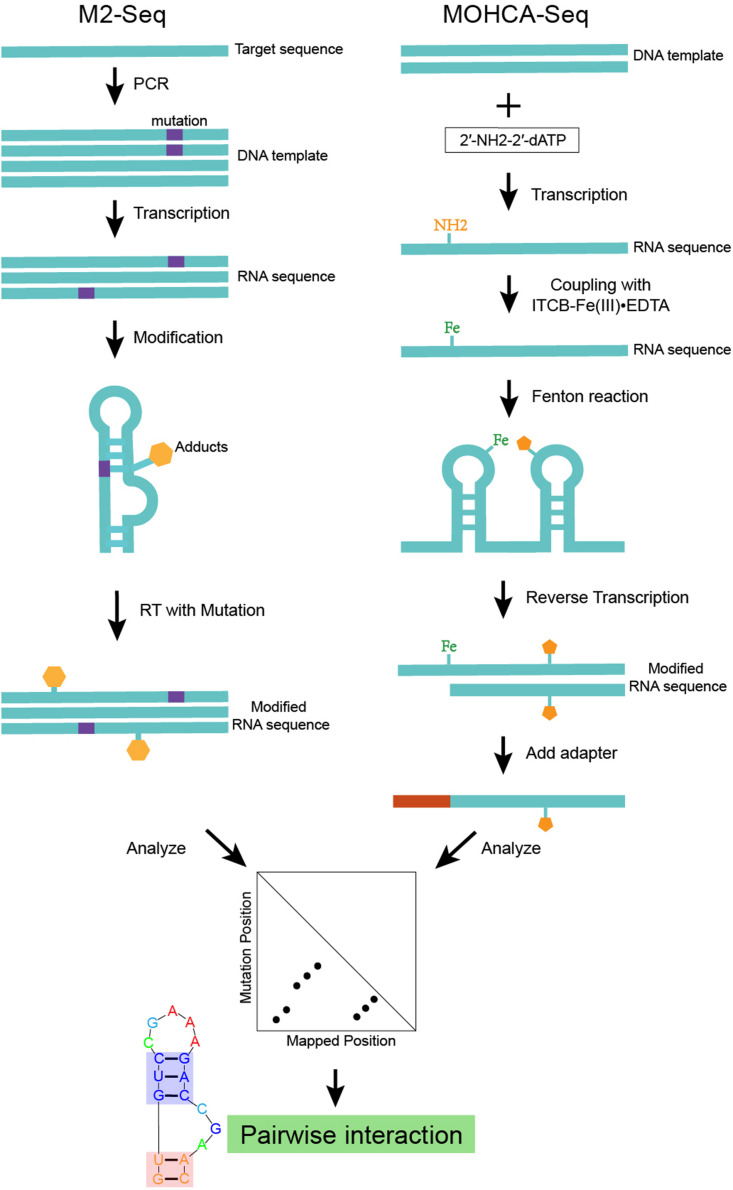
An example of structure modeling based on M2-seq (or MOHCA-seq). This scheme shows the workflow of M2-seq and MOHCA-seq experiments. Both approaches are based on the assumption that the mutated nucleotide in a base-pair tends to become more exposed and more detectable by chemical mapping. M2-seq uses DMS to probe the unpaired nucleotides introduced by error-prone PCR, while sequencing data can be analyzed by the M2-net algorithm based on the RT-stop mechanism. MOHCA-seq uses 2′-NH2-2′-dATP and isothio cyanobenzyl-Fe(III)EDTA to introduce Fe adducts into the RNA. While the Fenton reaction damages other nucleotides nearby the reacting Fe-modified nucleotide. Sequencing data analyzed by the MAPseeker algorithm highlights the proximally tertiary structure interactions.

M2-seq (Cheng et al., 2017), which is an advanced version of mutate-and-map (Figure 2), uses error-prone PCR to introduce mutations and couple DMS modification with HTS (high-throughput sequencing) based on the RT-mutate mechanism. M2-net is the algorithm to normalize M2-seq data and get secondary structure information (Cheng et al., 2017). MOHCA (Das et al., 2008) is an experiment that can map the positions of nucleotides that are close together in the three-dimensional structure. Highly reactive chemicals [2′-NH2-2′-dATP and isothio cyanobenzyl-Fe(III)EDTA] are attached to the nucleotides. And the Fenton reaction damages other nucleotides nearby the reacting Fe-modified nucleotide. It is possible to map the positions of these reacting nucleotides by detecting the damaged nucleotides. However, finding the damaged nucleotides is tedious and requires specialized equipment. MOHCA-seq (Cheng et al., 2015) is a new development of MOHCA (Figure 2), which couples MOHCA with HTS based on RT-stop. MAPseeker is the computational program used to analyze MOHCA-seq data and infer proximal tertiary interactions. Both the base-pair information from M2-seq and the tertiary interaction information from MOHCA-seq are key restraints for RNA structure modeling.

Besides *in vitro* structure probing, structure ensembles, and structure dynamics in living cells (Ganser et al., 2019) *in vivo* probing is being rapidly developed. Apart from *in vitro* structure probing, methods like DMS, SHAPE, and HRP can also be applied *in vivo* (Climie and Friesen, 1988; Moazed et al., 1988; Kwok et al., 2013; Spitale et al., 2013, 2015; Tyrrell et al., 2013; Ding et al., 2014; Smola et al., 2015a, 2016; Watters et al., 2016a; Lee et al., 2017; Feng et al., 2018; Mitchell et al., 2018). DMS demonstrates the advantages of its small size and short reaction time to capture some transient changes in structure (Waldsich, 2002; Waldsich et al., 2002; Tijerina et al., 2007). Reagent such as 1M7 (Tyrrell et al., 2013; McGinnis and Weeks, 2014; McGinnis et al., 2015) FAI, and NAI (Spitale et al., 2013) are developed to be used for *in vivo* SHAPE probing, while NAI shows the potential to reveal the structural differences between *in vivo* and *in vitro* probing (Kwok et al., 2013; Hector et al., 2014). Complementarily, HRP can probe RNA dynamics, folding (Latham and Cech, 1989) and solvent accessibility (Latham and Cech, 1989) in living cells (Kubota et al., 2015). More detailed information about *in vivo* probing and its challenges can be obtained from the review from Kubota et al. (2015). A recent study presented by Tomezsko et al. (2020) integrates DMS-MaPseq data with the DREEM (detection of RNA folding ensembles using expectation-maximization) algorithm to identify possible *in vivo* structures, suggesting a systematic method for dynamic detection and captures transient conformations *in vivo*. Another *in vivo* RNA–RNA interaction and RNA structure detection method, MARIO, utilizes an RNA linker to identify RNA–RNA interactome and finds secondary single-stranded regions as well as tertiary proximal contacts (Nguyen et al., 2016).

## Experimental Data-Driven Computational Modeling of RNA 3D Structures

### Types of Data-Driven Restraints

**FIGURE 3:**
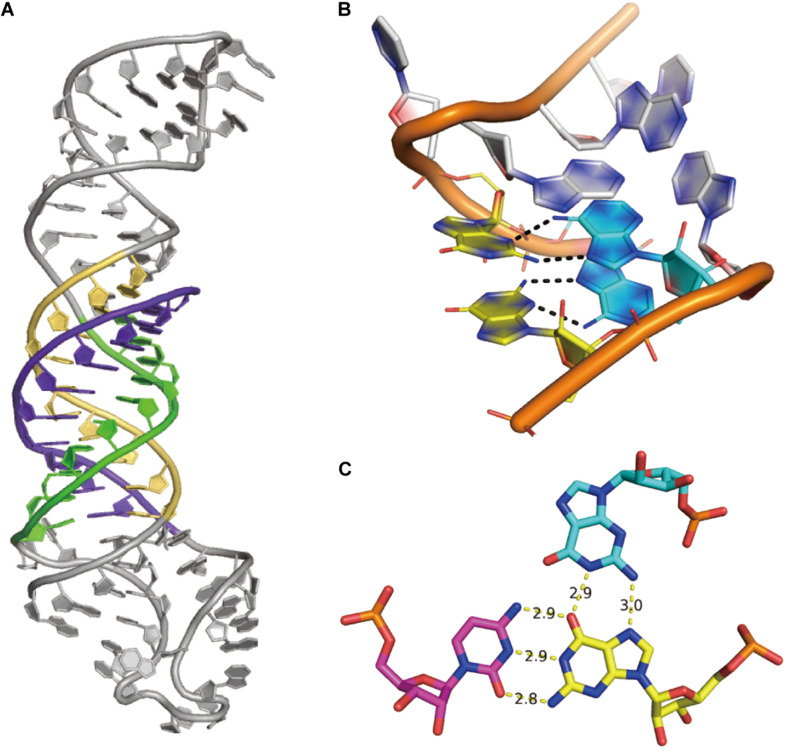
Examples of non-canonical interactions. **(A)** The UA-U-rich RNA triple helix in MALAT1_th11 RNA, PDB id 6SVS. **(B)** The simple k-turn with two *trans*-sugar-Hoogsteen G:A base pairs, PDB id 6HCT. **(C)** The structure of the G18 > G2:C39 triple interaction in glutamine-II riboswitch, PDB id 6QN3.

**FIGURE 4:**
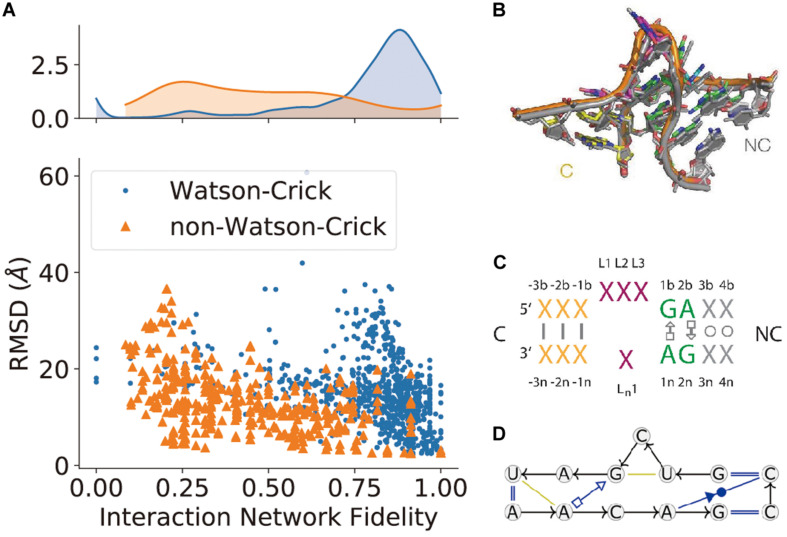
The prediction of non-canonical base-pairs. **(A)** The comparison between Watson–Crick and non-Watson–Crick base-pairs prediction in terms of interaction network fidelity (INF; Parisien et al., 2009) in RNA-Puzzles. **(B)** The superimposition of recurrent kink-turn modules. **(C)** The graphical abstraction of the kink-turn module. **(D)** The module abstraction in the *Basepairing* program.

Most experimental data alone, except x-ray crystallography and high-resolution cryo-EM, is not enough to infer atomic-resolution RNA structure. Hence computational modeling is indispensable in interpreting the experimental data into an atomic description of RNA 3D structure. Such computational modeling can be based on existing structure determination software, such as Phenix (Liebschner et al., 2019) for x-ray crystallography and cryo-EM, or CANDID (Herrmann et al., 2002) for NMR, or based on structure prediction programs, e.g., Rosetta (Das and Baker, 2007), Assemble2 (Jossinet et al., 2010; Jossinet, 2015), or MC-sym (Parisien and Major, 2008). The type of data used in structure modeling can be classified into five types:

(1) Sequence and sequence alignments are also determined from sequencing experiments. Sequence alignments can indicate certain co-evolution between base pairs. With the human genome project (Bentley, 2000) and advances in sequencing technologies (Schuster, 2008), a massive number of metagenome sequences are being deposited in databases (Sayers et al., 2011). Sequence covariation gives an alternative to understanding base-pair interactions and even RNA-protein interactions (Weinreb et al., 2016). The secondary structures of many significant RNA structures, such as the 16S ribosomal RNA structure (Noller and Woese, 1981), were derived from sequence covariations. Besides, sequence covariation also predicts some long-range Watson–Crick base-pairs in pseudoknots: a successful case was illustrated in the group I intron structure (Lehnert et al., 1996). DIRECT is a new algorithm reported to improve the prediction in long-range contacts and captures more tertiary structural information (Jian et al., 2019). A recent study demonstrated some success in RNA secondary structure prediction through the integration of sequence alignment based on direct coupling analysis and minimum free energy (MFE) (He et al., 2020). A machine learning approach was also reported to achieve better secondary structure prediction through the integration of direct coupling analysis and SHAPE data (Calonaci et al., 2020). However, some functional long non-coding RNAs may not necessarily have structures and may not have many homologous sequences or some sequence alignments could be too conserved to infer covariations. This severely limits the prevalence of these covariation-based RNA structure prediction methods. Details of covariation bases methods have been discussed by Rivas et al. (2017, 2020), while the resulting method has been used to curate Rfam RNA sequence families.
(2) The 3D shape of the molecule ranges from high-resolution x-ray crystallography to SAS, which only describes the global topology of a structure. In the case of x-ray crystallography and high-resolution cryo-EM, atomic coordinates may directly fit into the density maps. As for low-resolution density maps, computational modeling is required to generate structural models to fit in the global topology, while additional information (possibly the other three types of information, elaborated in the integrative modeling section below) may help the modeling.
(3) Features of a single nucleotide, such as paired/unpaired state and buried/exposed state, can be inferred from chemical probing data. These features are normally used to validate the secondary structure predicted by minimum free energy (MFE), e.g., using SHAPE to probe RNA secondary structures or to test probing profile change upon protein/ligand binding. Some of these HTS-based experiments, including DMS-seq (Rouskin et al., 2014) and SHAPE-seq (Loughrey et al., 2014; Watters et al., 2016b), have been commercialized. Together with these experiments, computational methods have been developed to derive more accurate structural information. An RNAsc method is proposed according to SHAPE data, which includes pseudo-energy terms and base stacking positions, making it more accurate to predict the secondary structure (Zarringhalam et al., 2012). Another method also utilizes pseudo-energy information obtained from chemical probing and integrates with thermodynamic folding algorithms to reach a good secondary structure prediction (Washietl et al., 2012). The Kalman approach is used to filter SHAPE data from noise (Vaziri et al., 2018) and the RING-MaP method reveals diverse interactions at both secondary and tertiary levels (Krokhotin et al., 2017). FoldAtlas was developed for high-throughput chemical probing data processing (Norris et al., 2017) and a statistical modeling was developed to improve the sensitivity of high-throughput probing data (Selega et al., 2017). An SNPfold algorithm was designed to recognize SNP-induced conformational changes in genome-wide analysis (Halvorsen et al., 2010). The above mentioned DREEM algorithm can be used to characterize different conformations from DMS-MaPseq data (Tomezsko et al., 2020). These examples show that efforts made in data processing modeling and methods may give us more useful and accurate information.
(4) Base pair interaction cannot only be inferred from sequence covariations (De Leonardis et al., 2015; Weinreb et al., 2016) but can also be determined from biochemical experiments (e.g., M2-seq) or secondary structure prediction (Zuker, 2003). Considering the base-pairing nature of RNA structures, this type of information has particular importance in determining the structure. In terms of secondary structure prediction, the MFE (minimum of free energy) structure simplifies the RNA structure as Watson–Crick base-pair interactions. Thus, it can be predicted by the combination of a loop-based energy model and the dynamic programming algorithm introduced by Zuker (2003). However, it ignores the contribution of pseudoknots and non-Watson–Crick interactions. When covariation or experimental evidence is available, the Watson–Crick base pairs in pseudoknots can still be correctly identified. It is most difficult to determine non-Watson–Crick base-pairs. The best way besides x-ray crystallography and cryo-EM is to use a combination of chemical probing experiments and sequence covariation. And some successful examples have been reported (Walczak et al., 1996). As discussed above, MOHCA-seq has demonstrated its ability in determining both Watson–Crick and non-Watson–Crick long-range base-pairs. In 3D structure modeling, the base-pair information is often used together with the features of a single nucleotide. For example, M2-seq is coupled with DMS to probe RNA structure and has been successful (Cheng et al., 2017).
(5) Non-canonical interaction-based RNA modules are RNA structural modules that are formed by non-canonical interactions and can be predicted by module prediction methods (Cruz and Westhof, 2011; Zirbel et al., 2015). Some recent determined RNA structures have demonstrated the importance of these non-canonical interactions (Butcher and Pyle, 2011): the recently crystalized MALAT1_th11 RNA (Ruszkowska et al., 2020) shows a UA-U-rich RNA triple helix with 11 consecutive base triples (Figure 3A); the two *trans*-sugar-Hoogsteen G:A base-pairs in the kink-turn module (Huang et al., 2019a) enables its folding in 3D (Figure 3B); and the triple interactions in the pseudoknot structure of the glutamine-II riboswitch (Huang et al., 2019b) is known to be crucial for ligand binding (Figure 3C). A structural module in RNA is a set of ordered *non*-Watson–Crick base-pairs embedded between Watson–Crick pairs, which are recurrent in the RNA structure (Figure 4B). With a good sequence alignment, such modules can often be predicted because of their restricted sequence variations. Automatic prediction programs based on this pragmatic approach have been developed (Cruz and Westhof, 2011; Theis et al., 2013). Without *non*-Watson–Crick interactions, the structural description is still at the secondary structure level. According to RNA-Puzzles, the prediction accuracy of *non*-Watson–Crick base-pairs is less accurate than the prediction of Watson–Crick base-pairs (Figure 4A), hindering the accurate RNA structure prediction. RNA structural modules are important for the folding of 3D RNA structures as well as for their functionality. For example, the kink-turn module, known to bind to the L7Ae protein (Turner et al., 2005), encodes a certain signature in its sequence (Figure 4C) and folds into a fixed structural fold (Figure 4B). Such a signature can be summarized by a probabilistic model (Cruz and Westhof, 2011; Sarrazin-Gendron et al., 2019), such as the *Basepairing* program (Figure 4D), or by machine learning (Zirbel et al., 2015).

### Types of Structure Prediction Approaches

**FIGURE 5:**
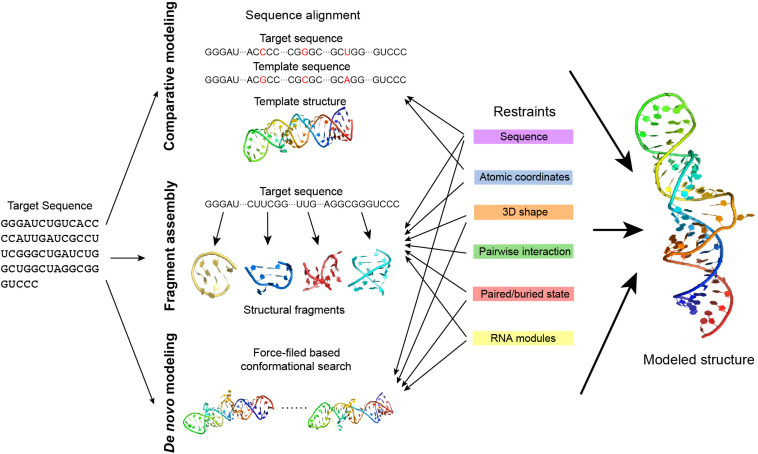
The approaches of RNA 3D structure modeling. Comparative modeling, fragment assembly, and *de novo* modeling are the basic approaches in predicting RNA structures. Comparative modeling is based on the availability of one or more homologous structures, while fragment assembly uses known structural fragments to assemble the structure. *De novo* modeling searches for the best conformation in the space considering the physical or empirical force-fields. Experimental data generates different types of restraints in structure modeling and can be applied in different modeling approaches. Details of the restraints and the related modeling approaches are explained in the main text.

All of these five types of structural information complement each other. Therefore, the integration of several types of the available information may improve the prediction of RNA structures. When neither a crystalline structure nor high-resolution cryo-EM data is available, the modeling of RNA structure in 3D may rely on RNA 3D structure prediction, which uses some existing knowledge about RNA structure derived from the existing databases. RNA 3D structure prediction has used some computational approaches, such as comparative modeling, fragment assembly, and *de novo* modeling (Figure 5) details can be referred to in the review paper by Rother et al. (2011a).

Comparative modeling is based on the assumption that homologous RNA molecules present similar structures in the relatively conservative regions. An RNA can be predicted using the known homologous structure in the database as a template. A prediction may either use the pairwise sequence alignment to model based on the template structure (Rother et al., 2011b) (ModeRNA) or translate the template structure into structural restraints in prediction (Flores et al., 2010) (RNABuilder). However, comparative modeling highly depends on the availability of a homologous template, thus it cannot be applied to explore a novel RNA structure.

Fragment assembly first decomposes the known RNA structures into fragments and makes a fragment library, and predicts an RNA structure by searching fragments that are similar to the target sequence and assembling them together. Several approaches in this type use secondary structure as a predefined restraint [e.g., RNAComposer (Popenda et al., 2012), 3dRNA (Zhao et al., 2012), and VfoldLA (Xu et al., 2019)], while experimental data may help in the determination of the secondary structure.

*de novo* modeling is a collective name for those predictions without templates. *de novo* modeling is normally based on force-field simulation and searches for the ideal structure by sampling the conformational space [e.g., NAST (Jonikas et al., 2009), iFoldRNA (Sharma et al., 2008) (Krokhotin et al., 2015), and SimRNA (Boniecki et al., 2016)]. As the whole conformational space can be too large to explore, using predefined restraints such as the secondary structure to narrow down the search space may effectively reduce the unnecessary search and achieve a better prediction.

### Integrative Modeling

When no homologous template is available, fragment assembly and *de novo* modeling may integrate various types of information to help with the prediction. A widely-used approach is to predict the secondary structure first and predict the 3D structure according to the secondary structure. One simple case is the RNAComposer: one can use RNAfold from ViennaRNA (Lorenz et al., 2011) to predict the secondary structure and use RNAComposer to assemble the 3D modeling according to the predicted secondary structure using the fragment library RNA FRABASE (Popenda et al., 2010). A more complicated version could use DMS-seq and M2-seq to constrain the secondary structure, while use MOHCA-seq to probe the tertiary interactions. Then, use fragment assembly to build an initial 3D model and optimize it through conformational search using Rosetta or other force-field based methods. One may even use covariations from the multiple sequence alignment to confirm the base-pairs/interactions, and use module prediction methods to assign the non-canonical base-pairs. Such modeling integrates several types of information (features of a single nucleotide, base-pair interaction, and non-canonical interactions) in several prediction methods (fragment assembly and *de novo* modeling). Normally, integrative modeling is defined as modeling that uses more than one modeling approach. To extend, it may also integrate different types of information in different types of prediction methods to achieve a high-quality prediction. As experiments, especially the HTS based techniques, are becoming better established and cheaper, integrative modeling using experimental data is becoming easier and makes modeling more reliable.

Various integrative approaches have been reported in recent years: A fully computational workflow, EvoClustRNA (Magnus et al., 2019), effectively integrates *de novo* modeling and fragment assembly and statistical potential to achieve accurate predictions. EvoClustRNA first selects a set of sequences homologous to the target sequence to be modeled and uses both FARFAR (fragment assembly) and SimRNA (*de novo* modeling) to model all the sequences. The conserved regions of all these sequences are extracted and clustered. And the predicted structure is selected from the most commonly preserved structural arrangements of the homologous structures based on a statistical potential. Although EvoClustRNA is a fully computational workflow, it demonstrates good predictions in several targets of RNA-Puzzles. The integrative modeling platform (Russel et al., 2012) is a computational platform to integrate SAS, EM, x-ray crystallography, or NMR data by comparative modeling. It was designed for protein structures but may also extend to other macromolecules like RNA. Another example is PLUMED-ISDB, which is based on the molecular dynamics library PLUMED (Tribello et al., 2014). It may integrate experimental data from NMR, FRET, SAXS, or cryo-EM to model the structure and dynamics of RNA as well as other biomacromolecules (Bonomi and Camilloni, 2017). Apart from the above mentioned approaches, there are also other computational modeling methods, such as molecular dynamics (MD) and quantum mechanics (QM). Discussion about those methods have been introduced in other reviews (Dawson and Bujnicki, 2016; Miao and Westhof, 2017; Schlick and Pyle, 2017).

However, not all experimental data are accurate and informative enough. Integrating noisy or bad data into structure modeling may result in a worse prediction. A recent study (Wang et al., 2019) performing structure prediction with excessive restraints from experimental data demonstrated this conclusion. Therefore, more strict assessment and calibration of the experimental data for modeling is needed and the related computational methods need to be developed.

## Recent Successes in Computation-Aided RNA Structure Determination

Massive progress is being made through the integration of different methods, both computational and experimental. Not only experimental data can be used as restraints to help computational modeling, but also computational approaches benefit experimental research.

One such advance was to use the *de novo* predicted structure to determine the x-ray crystal phase (Huang et al., 2019b). Huang et al. (2019b) reported *de novo* predictions, which are within 3 Å root mean square deviation (RMSD) from the crystal structure of glutamine-II riboswitch. And two of the predicted models were able to achieve the molecular replacement to determine the phase of the crystal. This work reports a useful potential that computational modeling may take up a more important role in interpreting experimental data.

With the rapid development of cryo-EM technology, computational studies are accentuated to help in structure determination. Deep learning models have been proposed to address the laborious particle picking problem (Norousi et al., 2013; Wang et al., 2016; Al-Azzawi et al., 2019; Yao et al., 2019). For high-resolution cryo-EM density maps, a fully automatic computational method, map_to_model (Terwilliger et al., 2018) from Phenix (Liebschner et al., 2019), is capable of yielding high-accuracy initial models. However, it is still not easy to deal with low-resolution data. And more efforts are being made. DRRAFTER (Kappel et al., 2018) allows for the tracing of RNA atomic coordinates in the biologically important but low-resolution regions using *de novo* computational modeling. cryo-EM maps offer the information of the 3D shape of a molecule, while computational modeling infers the atomic coordinates from this information.

**FIGURE 6:**
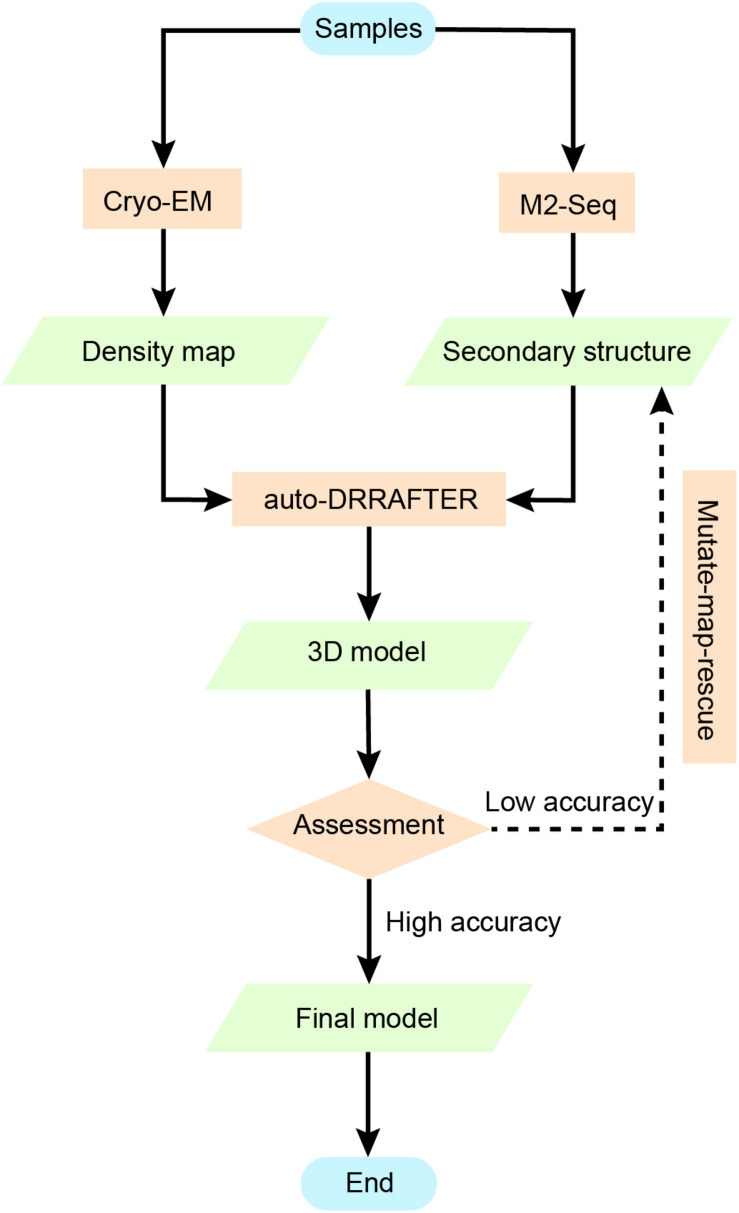
Using chemical mapping-based structure modeling to model low-resolution cryo-EM data (Ribosolve). The Ribosolve approach is a recent example of integrative modeling using moderate-resolution cryo-EM maps, chemical mapping, and Rosetta computational modeling. M2-seq probes the base-pair interactions, while cryo-EM restrains the three-dimensional shape of the structure topology. Force-field-based modeling optimizes the structure based on the learned knowledge of the force-field derived from known RNA structures. The generated models are assessed by modeling convergence, while mutate-map-rescue provides an alternative to optimize the secondary structure information and improve the modeling.

However, as DRRAFTER was developed specifically for modeling large RNA-protein complexes, which require initial manual setup, it may not be effective and may include bias when modeling smaller RNAs without protein partners. auto-DRRAFTER was designed to address this problem in an automatic way. auto-DRRAFTER constitutes the computational part of Ribosolve (Kappel et al., 2020), which is a hybrid workflow integrating moderate-resolution cryo-EM maps, chemical mapping, and Rosetta (Das and Baker, 2007) computational modeling. As shown in Figure 6, Ribosolve first uses native gel to check for the formation of sharp bands in the RNA samples. RNA secondary structures can be determined by M2-seq, while the 3D shape of the RNA global architecture can be obtained from cryo-EM. In addition, mutate-map-rescue experiments can confirm or refine novel secondary structure rearrangements. Based on these two types of information, auto-DRRAFTER is able to build all-atom models. Model accuracy is predicted from the overall modeling convergence, while uncertain regions are identified by comparing the per-residue-convergence and real-space correlation between the map and model. As a result, Ribosolve dramatically captures the structure of full-length *tetrahymena* ribozyme and builds 13 RNA structures without protein binding ranging from 119 to 338 nt. Moreover, the structure of several other important RNA, like hc16 ligase and full-length *Vibrio cholerae* and *Fusobacterium nucleatum* glycine riboswitch aptamers, are determined. The SAM-IV riboswitch structure illustrates Ribosolve’s ability in solving small RNA structures within 40 kDa (Zhang et al., 2019). These results from Ribosolve demonstrate a rapid and routine determination of RNA-only 3D structures.

There are also other successful examples in both computational-aided structure determination and in new computational modeling methods. Zhang et al. (2018) integrated NMR and cryo-EM data using MD simulation to solve the 30 kDa HIV-1 RNA. NMR data and SAXS data were used jointly to determine the HIV-1 intron splicing silencer structure (Jain et al., 2016). Ab initio 3D structure modeling helped in determining the HIV-1 Rev response element using SAXS data (Fang et al., 2013). The RS3D method can utilize secondary structure information, SAXS data, and any tertiary contacts information to determine the RNA structure topology (Bhandari et al., 2017). FARFAR (Das et al., 2010; Yesselman and Das, 2016) and FARFAR2 (Watkins et al., 2020) were developed to model non-canonical RNA structure at near-atomic accuracy. Swellix explores RNA conformational space when integrating data from crystallography, cryo-EM, or *in vivo* crosslinking and chemical probing methods (Sloat et al., 2017). Statistical modeling helped to construct RNA structure landscapes from structure profiling data, which can facilitate the studies of RNA dynamics and function (Li and Aviran, 2018).

## Challenges and Perspectives

In spite of the good number of RNA structures being determined, it is still urgent to probe the structural basis of RNA functions. For example, with the break out of virus infection [2015–2016 Zika virus (Akiyama et al., 2016), 2019 coronavirus (Huang et al., 2020)], rapid determination of the structures of functional elements in virus RNA may provide invaluable insights into human health. Computational modeling is playing a more critical role in biological studies (Markowetz, 2017), while RNA structure determination is a particular field where computational modeling can exert its potential. Although RNA structure determination is now becoming rapid and routine, there exist several major challenges.

A major challenge is to improve experimental accuracy for high-throughput biochemical experiments. For this purpose, it is necessary to systematically benchmark these high-throughput techniques with high-resolution RNA structures. However, when high-resolution RNA structures are not available, such benchmarking of experimental accuracy is indirect and confounded with computational modeling. Thanks to the increasing throughput of experimental data being generated, a more realistic evaluation of experimental noise will become possible. Considering that the high-throughput sequencing data tends to reflect a mixture of the structural conformations, computational modeling methods are expected to deconvolve this structural information and dissect the conformations. High-throughput experiments will also help in determining more unknown structures in Rfam families.

Another significant challenge lies in probing RNA structures *in vivo* to understand the structural dynamics. Although DMS, SHAPE, HRP as well as other recent methods, such as DMS-MaPseq, provide good tools for structure ensembles and dynamics detection, the folding process to the functional state remains unsolved. Compared to biochemical experiments, x-ray crystallography and cryo-EM normally need to prepare RNA samples in extreme conditions which are relatively difficult. And high resolution methods with easier sample preparation may be developed in the future (Stagno et al., 2017). Computational modeling is the interface between experimental data and the RNA structure coordinates. It is always a central problem to improve computational modeling according to the experiments and also help to correct experimental biases.

In terms of structure modeling, it is known that the prediction of non-canonical interactions (non-Watson–Crick base-pairs) is still difficult. A good description of these non-canonical interactions both benefit the RNA structure modeling and the comprehension of functional RNA modules. To improve the RNA structure prediction methods, optimizing non-canonical interaction is still inevitable. As non-Watson–Crick base-pairs could include additional chemical probing information, e.g., the N7G methylation by DMS, new computational methods dealing with these different chemical probing datasets may effectively improve the determination of non-Watson–Crick interactions and achieve better structure predictions. Automatic modeling workflow is another challenge in RNA modeling. Most currently available automatic web servers depend on the sole input of the RNA sequence and optionally the secondary structure information. And the structure modeling accuracy is not ideal because of the lack of experimental data. However, it will be key to transform various experiment data into structural restraints in a standard manner. Automatic web servers should also promote the input of experimental data as restraints to improve prediction accuracy.

With the increasing number of RNA structures being solved, our knowledge of RNA structure will be enlarged. More new RNA folds will be determined, and more structural modules will be explored. Even more structural rules, such as the coaxial rule, will be discovered. Gaining more knowledge about RNA structure will allow for better force-fields, better structure modeling, and a better understanding of the RNA functions. The increasing number of experimentally determined structures in the PDB database will expand the knowledge of existing RNA structure fold databases, e.g., MC-fold (Parisien and Major, 2008) and RNA FRABASE (Popenda et al., 2010), and improve the structure prediction and modeling.

## Author Contributions

ZM designed the project. BL drafted the manuscript. All authors contributed to the manuscript.

## Conflict of Interest

The authors declare that the research was conducted in the absence of any commercial or financial relationships that could be construed as a potential conflict of interest.
